# Mechanism of Action of Isoflavone Derived from Soy-Based Tempeh as an Antioxidant and Breast Cancer Inhibitor via Potential Upregulation of miR-7-5p: A Multimodal Analysis Integrating Pharmacoinformatics and Cellular Studies

**DOI:** 10.3390/antiox13060632

**Published:** 2024-05-22

**Authors:** Fahrul Nurkolis, Nurpudji Astuti Taslim, Dain Lee, Moon Nyeo Park, Seungjoon Moon, Hardinsyah Hardinsyah, Raymond Rubianto Tjandrawinata, Nelly Mayulu, Made Astawan, Trina Ekawati Tallei, Bonglee Kim

**Affiliations:** 1 Department of Biological Sciences, Faculty of Sciences and Technology, State Islamic University of Sunan Kalijaga (UIN Sunan Kalijaga), Yogyakarta 55281, Indonesia; fahrul.nurkolis.mail@gmail.com; 2 Division of Clinical Nutrition, Department of Nutrition, Faculty of Medicine, Hasanuddin University, Makassar 90245, Indonesia; 3 Department of Pathology, College of Korean Medicine, Kyung Hee University, Seoul 02447, Republic of Korea; 4 Division of Applied Nutrition, Department of Community Nutrition, Faculty of Human Ecology, IPB University, Bogor 16680, Indonesia; 5 Department of Biotechnology, Faculty of Biotechnology, Atma Jaya Catholic University of Indonesia, Jakarta 12930, Indonesia; 6 Department of Nutrition, Faculty of Health Science, Muhammadiyah Manado University, Manado 95249, Indonesia; 7 Department of Food Science and Technology, Faculty of Agricultural Engineering and Technology, IPB University, Jl. Raya Dramaga, Bogor 16680, Indonesia; 8 Department of Biology, Faculty of Mathematics and Natural Sciences, Universitas Sam Ratulangi, Manado 95115, Indonesia; 9 Korean Medicine-Based Drug Repositioning Cancer Research Center, College of Korean Medicine, Kyung Hee University, Seoul 02447, Republic of Korea

**Keywords:** tempeh, soybean, anticancer, antioxidants, functional food, miR-29a-3p, in silico, in vitro

## Abstract

Breast cancer presents a significant global health challenge with rising incidence rates worldwide. Despite current efforts, it remains inadequately controlled. Functional foods, notably tempeh, have emerged as promising candidates for breast cancer prevention and treatment due to bioactive peptides and isoflavones exhibiting potential anticancer properties by serving as antioxidants, inducing apoptosis, and inhibiting cancer cell proliferation. This study integrates pharmacoinformatics and cellular investigations (i.e., a multifaceted approach) to elucidate the antioxidative and anti-breast cancer properties of tempeh-derived isoflavones. Methodologies encompass metabolomic profiling, in silico analysis, antioxidant assays, and in vitro experiments. Daidzein and genistein exhibited potential therapeutic options for breast cancer treatment and as antioxidant agents. In vitro studies also supported their efficacy against breast cancer and their ability to scavenge radicals, particularly in soy-based tempeh powder (SBT-P) and its isoflavone derivatives. Results have demonstrated a significant downregulation of breast cancer signaling proteins and increased expression of miR-7-5p, a microRNA with tumor-suppressive properties. Notably, the LD_50_ values of SBT-P and its derivatives on normal breast cell lines indicate their potential safety, with minimal cytotoxic effects on MCF-10A cells compared to control groups. The study underscores the favorable potential of SBT-P as a safe therapeutic option for breast cancer treatment, warranting further clinical exploration.

## 1. Introduction

Breast cancer is a global health concern, with increasing prevalence rates worldwide. According to cancer mortality profiles, the most common new malignancies in Indonesia are lung (23.1%), prostate (30.7%), and breast (43.3%) cancers [1]. The American Cancer Society estimates 300,590 new cases and 43,700 deaths from breast cancer in 2023 [2]. In Brazil, the latest incidence of breast cancer in 2023 rose by 29.7% compared to the previous three years [3]. Breast cancer is a complex disease with important genetic, environmental, and lifestyle or behavioral components. The risk factors include genetic factors, particularly family history, diet, obesity, smoking, alcohol consumption, ionizing radiation exposure, and residual factors such as menstruation, childbirth, and lactation [4]. According to recent data, breast cancer is one of the most common cancers among women in developed and developing countries [5]. Current approaches to breast cancer management involve various strategies ranging from early detection screenings to more advanced therapies, such as chemotherapy, radiotherapy, and targeted therapy [6,7]. However, these efforts have not optimally prevented and addressed breast cancer.

Functional foods, especially tempeh, have garnered attention in the context of breast cancer prevention and treatment. Tempeh is mostly made up of bioactive peptides and isoflavones, which may help prevent cancer by acting as antioxidants, apoptosing cancer cells, and inhibiting the growth of cancer cells [8]. Tempeh, a traditional soybean fermentation product, is widely known for its rich nutritional content, including isoflavones and their bioactive compounds [9]. Phenolic compounds, saponins, phytic acid, and enzyme inhibitors like trypsin and Bowman–Birk inhibitors are associated with soybean anticancer properties; however, isoflavones, soybean phytochemicals with antioxidant properties that can shield human cells from oxidative stress linked to cancer, are the most noteworthy [10,11]. Genistein possesses antioxidant and anticancer activities, particularly by regulating specific gene expression [12]. Additionally, genistein, the main soy isoflavone, inhibits the growth, metastasis, and development of cancer in animal models, especially by modifying the genes involved in apoptosis and cell cycle regulation [13]. However, despite research highlighting tempeh’s antioxidant and anticancer potential, studies integrating the mechanisms of action of its isoflavone compounds are still limited. Specifically, research connecting tempeh isoflavones with the regulation of specific microRNA expression, such as miR-7-5p, is lacking in breast cancer prevention and treatment.

This study aims to fill this knowledge gap by integrating pharmacoinformatics and cellular studies to understand the mechanism of action of tempeh-derived isoflavones as antioxidants and breast cancer inhibitors, particularly through miR-7-5p upregulation. The findings from this research should complement our understanding of the health properties of tempeh and significantly contribute to the literature regarding breast cancer.

## 2. Materials and Methods

### 2.1. Apparatus and Materials

The materials and apparatus used in this study are listed according to research stage, including cleaning, drying, determining metabolite profiles, and in silico and in vitro testing. The apparatus includes a drying oven (Memmert Incubator IN55, Schwabach, Germany), rotary evaporator (Merck KGaA, Darmstadt, Germany), blender (CosmosBlender; Tangerang, Indonesia), binocular microscope, spectrophotometer (SmartSpec Plus, Bio-Rad Laboratories. Inc., Hercules, CA, USA), and temperature-functional centrifuge (Eppendorf, Hamburg, Germany).

The materials used in this study include 96% ethanol, acetonitrile, methanol, formic acid, trypsin-ethylenediaminetetraacetic acid solution, MCF-7 and MCF-10A, potassium persulfate, sodium chloride, ABTS, mitoxantrone and talazoparib (Sigma-Aldrich, Darmstadt, Germany), Dulbecco’s modified eagle medium (DMEM), bovine serum albumin (BSA), penicillin and streptomycin (Thermo Fisher Scientific, Waltham, MA, USA), FRAP (BioVision, Milpitas, CA, USA), STAT3, EGFR, IGF1R kinase, CDK1, ABCC1, TGFBR1/ALK5, PARP1, ROS1 kinase, and iNOS from Elabscience (Wuhan, China). Genistein, daidzein, and zearalenone were provided by Sigma-Aldrich (Darmstadt, Germany).

### 2.2. Soy-Based Tempeh Powder Preparation

**Figure 1:**
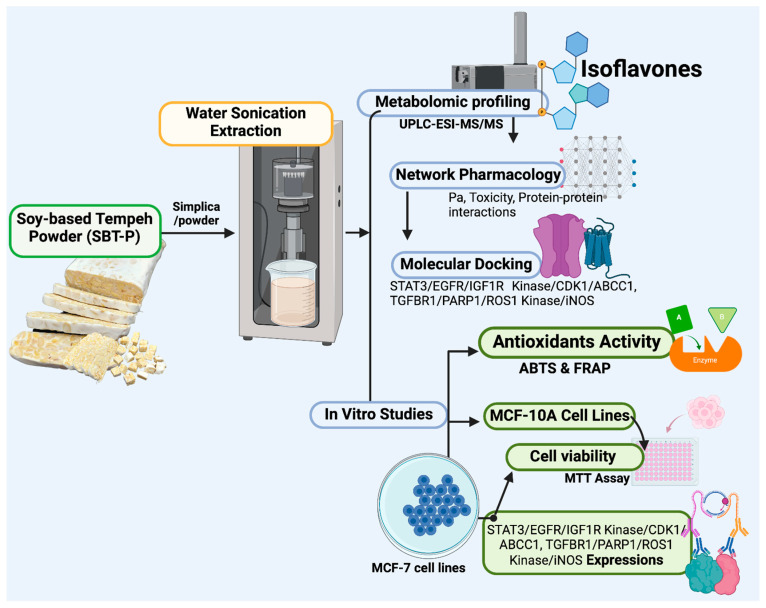
Schematic of SBT-P study workflow.

Samples of soy-based tempeh powder (SBT-P) were obtained online from Sanfood Indonesia (Tangerang, Indonesia). All methods follow relevant guidelines and regulations for in vitro and plant studies. Sample size reduction was conducted with a blender to provide a coarse simplicia powder (Figure 1). This study aims to see the benefits of SBT-P the way it is usually consumed (no chemical solvents) to examine its direct impacts: therefore, SBT-P was sonicated for 30 min (40 °C) using an ultrasound sonicator Branson 2510 model (400 W; St. Louis, MO, USA) with ultrapure water solvents and a ratio based on internal standards (1:4 *w*/*v*) (Figure 1). Due to SBT-P’s edible and resistant nature, sonicated samples were stored in aluminum foil at room temperature before further laboratory testing.

### 2.3. Phytochemical Profiling via Untargeted Metabolomic Profiling

Metabolomic profiling analysis and compound identification were conducted through similar research protocols [14]. SBT-P samples (50 mg) were weighed precisely into a tube, 80% methanol (800 μL) was added, and the mixture was vortexed for 90 s. Then, samples were stored at −40 °C for 1 h, vortexed for 30 s, held for 30 min, and centrifuged at 12,000 rpm for 15 min at 4 °C. Finally, 200 μL of supernatant was transferred into a vial for liquid chromatography–mass spectrometry (LC-MS) analysis. Ultra-performance LC-tandem MS (UPLC-MS/MS) was performed using an Ultimate 3000LC combined with Q Exactive MS (Thermo Fisher, Waltham, MA, USA) with electrospray ionization MS (ESI-MS) and an ACQUITY UPLC HSS T3 column with 100 mm × 2.1 mm (1.8 μm). The mobile phase (mp) comprised solvent A with 0.05% CH_2_O_2_ or formic acid–water and the mp of solvent B with acetonitrile (C_2_H_3_N via gradient elution (0–1.0 min, 95% A; 1.0–12.0 min, 95–5% A; 12.0–13.5 min, 5% A; 13.5–13.6 min, 5–95% A; 13.6–16.0 min, 95% A). The mobile phase flow rate was 0.3 mL/min. The column temperature was maintained at 40 °C, and the sample manager temperature was set at 4 °C. The sample injection volume was 40 μL. MS parameters were in positive ion (ESI+) and negative ion (ESI−) modes. 

### 2.4. In Silico Study

#### 2.4.1. Prediction of Bioactive Compound Activities and Druglikeness

The compounds identified in SBT-P were analyzed by the WAY2DRUG PASS prediction tool for their potential bioactivity or Pa (probability of being active) for treatment related to cancer, which specifically targets human breast cancer genes and proteins via analyzing structure–activity relationships (SARs). Input compounds were compared with known compounds with specific potency [15]. The Pa value represents the output prediction score obtained from the web (http://www.pharmaexpert.ru/passonline/predict.php; accessed on 20 March 2024), which indicates the potency of the tested compound; it reflects the accuracy of the prediction function obtained. A Pa > 0.4 indicates a high anticancer potential, and this study was limited to Pa > 0.7. Furthermore, the druglikeness analysis represents a series of pharmacokinetic parameters essential in the development of drugs by assessing a drug’s potential toxicity using the Protox II database (https://tox-new.charite.de/protox_II/index.php?site=compound_input; accessed on 20 March 2024). Drug similarity characteristics were determined for each ligand based on Ro5 or Lipinski’s rule of five, which was analyzed by the ADMETLab 2.0 database (https://admetmesh.scbdd.com/service/evaluation/index; accessed on 20 March 2024) using the SMILES notation of each compound as input [16,17,18]. The simplified molecular input line entry system notation, or SMILES, for SBT-P isoflavone was obtained from PubChem (https://pubchem.ncbi.nlm.nih.gov; accessed on 20 March 2024), and the data are displayed in Supplementary Table S1.

#### 2.4.2. Protein Target Identification and Analysis

Target analysis was performed for SBT-P by putting the SMILES notation for each identified element into the SuperPred target analysis program (https://prediction.charite.de; accessed on 20 March 2024). The cut-off values for accuracy and probability were set at 80% [19,20]. The genes and proteins were extracted from the Open Targets database (http://www.opentargets.org; retrieved on 20 March 2024) and linked to breast cancer and oxidants. A Venn diagram was then used to map the intersection between targets connected to diseases (breast cancer and oxidants) and those related to SBT-P. The DAVID website (https://david.ncifcrf.gov; accessed on 20 March 2024) was used to annotate SBT-P targets, with an emphasis on biological processes and Kyoto Encyclopedia of Genes and Genomes (KEGG) pathways [21].

#### 2.4.3. Network Pharmacology Analysis

The Search Tool for Retrieval of Interacting Genes/Proteins (STRING) database 12.0 was used to analyze the association between the target proteins of SBT-P and breast cancer [22]. The STRING proteins linked to breast cancer, including HIF1A regulators strongly correlated with breast cancer incidence, were included in the input, along with SBT-P target proteins and oxidants. The organism used in the STRING database study was *Homo sapiens*, and a high confidence score criterion of 0.90 was adopted to guarantee robust interactions. The final data were downloaded and displayed in TSV format. CytoScape 3.10.1 was then used for data processing for a thorough network analysis, study, and exploration of important network metrics, such as degree, betweenness centrality, and closeness centrality between signaling proteins [23].

#### 2.4.4. Molecular Docking Simulation

CB-Dock2, an enhanced version of the CB-Dock server for protein–ligand blind docking, was utilized to direct the cavity-detection-guided blind docking simulation. Cavity identification, docking, and homologous template fitting are all included in this technique. The protocols described in earlier articles were adhered to by the docking protocol (https://cadd.labshare.cn/cb-dock2/index.php; accessed on 21 March 2024). CB-Dock2 is a protein–ligand docking technique that uses CB-Dock2 to perform molecular docking and automatically finds binding sites, determines their center and size, and adjusts the docking box size based on the query ligands. CB-Dock streamlines the docking process and increases accuracy by predicting the binding sites of target proteins using the curvature-based cavity detection technique (CurPocket) and the binding poses of query ligands using CB-Dock2 (https://cadd.labshare.cn/cb-dock2/index.php; accessed on 21 March 2024). Moreover, the highest degree of centrality discovered in the reported signaling proteins—including those connected to their signaling pathways—was utilized for additional molecular docking analysis. 

The enzymes or proteins utilized include STAT3 (PDB ID: 1BG1), EGFR (1M17), IGF1R kinase (3LWO), CDK1 (6GU7), ABCC1 (2CBZ), TGFBR1/ALK5 (5QIK), PARP1 (4R6E), ROS1 kinase (3ZBF), and iNOS (3E7G), sourced from the RCSB protein databank. Before docking, water molecules and other heteroatoms were removed from the protein structures through the CB2-Dock server’s default settings. All target proteins were in .pdb format sourced from RSCB Protein Data Bank (https://www.rcsb.org; accessed on 20 March 2024). Ligands were acquired from PubChem in .sdf format (https://pubchem.ncbi.nlm.nih.gov; accessed on 20 March 2024), and the compounds unavailable in PubChem were visualized using ChemDraw MacBook 22.2.0.

### 2.5. Antioxidant Activity against ABTS and FRAP

The antioxidant activity was evaluated using potassium persulfate (K_2_S_2_O_8_, 2.4 mM) and ABTS (7 mM) combined in equal proportions (1:1). The mixture was shielded from light using aluminum foil and incubated at 22 °C for 14 h in the dark. The mixture was diluted to obtain a working solution (e.g., 1 mL of stock solution plus 60 mL of Ethanolo or EtOH) with an absorption of 0.706 (734 nm). A new working solution was prepared for each test. The SBT-P, genistein, daidzein, and zearalenone samples were stored at 35, 70, 105, 140, and 175 μg/mL to be diluted with the ABTS working solution (1 mL), and their absorbance was measured at 734 nm after 7 min. The ABTS inhibition is expressed as a percentage (%) and the formula to determine the value is as follows:

(1) $$Inhibition\;Activity\left(\%\right)=\left[\frac{A0-A1}{A0}\right]\times 100\%$$

where **A0** is the blank absorption and **A1** the standard or sample absorption.

The FRAP test was conducted using a previously published method [24]. Then, 300 mM of sodium acetate buffer (pH 3.6, 10 mL) was added to a 10 mM TPTZ solution in 40 mM of hydrochloric acid (1 mL) and 20 mM of ferrous (III) chloride (1 mL) as FRAP reagents. They were placed in water baths at 37 °C. The SBT-P, genistein, daidzein, and zearalenone samples, at 35, 70, 105, 140, and 175 μg/mL, were mixed with 1 mL (FRAP) reagents. Their absorbance was determined immediately at 593 nm. The FRAP values were recorded with the following equation: (***Ac***) is the absorbance of trolox or positive control after being reacted with FRAP reagent (as is the sample absorbance), and ***Ab*** is the absorbance of the blank reacted with distilled water and FRAP reagent.

(2) $$FRAP\;Value\left(\%\right)=\left[\frac{Ac-Ab}{Ac-Ab}\right]\times 2$$

### 2.6. In Vitro Study on Cancer Cell Lines

Human breast cancer cells (MCF-7 line) and normal breast epithelial cells (MCF-10A line) were obtained from the Laboratory of Biochemistry and Biomolecular in the Faculty of Medicine, Brawijaya University Malang (Indonesia). MCF-7 and MCF-10A cells were cultured in 96-well plates containing DMEM with 10% FBS and 1% antibiotic (100 UI/mL penicillin and 100 μL/mL streptomycin). Once the cultured cells reached 80% density, the cells were incubated at 5% CO_2_ and 37 °C. Cells were harvested periodically using a solution of trypsin–ethylenediaminetetraacetic acid.

#### 2.6.1. Protein and miR-7-5p Expressions 

STAT3, EGFR, IGF1R kinase, CDK1, ABCC1, TGFBR1/ALK5, PARP1, ROS1 kinase, and iNOS protein expressions were analyzed in vitro following the kit protocols and established experimental guidelines. The polyvinylidene difluoride (PVDF) membrane was treated with a blocking solution of skimmed dry milk at 5% in a T-TBS saline buffer with pH 7.4 (20 mmol/L Tris-HCl, 0.1% Tween 20, 0.138 mol/L NaCl) to detect STAT3, EGFR, IGF1R kinase, CDK1, ABCC1, TGFBR1/ALK5, PARP1, ROS1 kinase, and iNOS and prevent the membrane from absorbing any detection reagents. The membrane was treated with a blocking solution of 5% BSA in T-TBS to identify phosphorylated STAT3, EGFR, IGF1R kinase, CDK1, ABCC1, TGFBR1/ALK5, PARP1, ROS1 kinase, and iNOS exposed to PA (primary antibodies), followed by peroxidase with associated secondary antibodies by diluting antibodies in 5% BSA in T-TBS. The study aims to gain insight into protein expression by adopting this comprehensive antibody-based technique while ensuring precision through appropriate incubation conditions and antibody dilution. 

The experimental process involved seeding 5,000 MCF-7 cells into each well using 100 μL/well and treating with SBT-P, genistein, daidzein, and zearalenone at 74.88 μg/mL (the ABTS EC_50_) for 24 h. The data were analyzed to ascertain the percentage value relative to the control group (non-treated cells), which was facilitated through optical density (OD) measurements using a spectrophotometer at 620–665 nm.

In the MCF-7 line (treated and control), total RNA was extracted according to the manufacturer’s instructions with TRIzol reagent (Invitrogen Life Technologies, Waltham, MA, USA) and quantified at 260 nm. Experiments were conducted in triplicate. The cDNA was synthesized from 2 μg of total RNA using a miScript II RT Kit (QIAGEN, Hilden, Germany). Stem-loop RT primers were used for reverse transcription, and the obtained cDNA was used as a template to detect miR-7-5p expression in MCF-7. Quantitative real-time PCR (qRT-PCR) was performed using FastStart Essential DNA Green Master (Roche, Basel, Switzerland), referring to the manufacturer’s instruction protocol. Data were analyzed with the 2^−ΔΔCt^ method. 

#### 2.6.2. MTT Assay 

The cytotoxicity tests of cells MCF-7 and MCF-10A were conducted via the MTT method according to a previously published method [25]. MCF-7 and MCF-10A cells were incubated in 96-well plates for 24 h and administered with SBT-P, genistein, daidzein, and zearalenone at 0, 35, 70, 105, 140, and 175 μg/mL. According to comparable studies, mitoxantrone and talazoparib were applied as positive controls [25]. SBT-P, genistein, daidzein, zearalenone, and positive controls were added and incubated for 24 h. We isolated the cell line using PBS 1× liquid and incubated at 37 °C together with 0.5 mg/mL (100 μL) MTT. Then, 100 μL of stopping reagent, namely DMEM, was given to each well plate after 30 min. The absorbance of the samples was measured using a microplate reader (550 nm). Three triple trials were performed to minimize bias for each treatment group or sample. The eligible cells are presented as percentages with the following formula:

(3) $$Percentage\;of\;Living\;Cells\;or\;Viability\left(\%\right)=\frac{A-B}{C-B}$$

where **A** is the treated cell absorbance, **B** is the absorbance of blank samples, and **C** is the control cell absorbance.

### 2.7. Data Analytics and Management 

Statistical analysis was conducted using GraphPad Prism Premium 10 (GraphPad Software, Inc., San Diego, CA, USA). The Shapiro–Wilk test was performed to evaluate the data distribution. If the data were normally distributed (significance < 0.05), a one-way ANOVA was performed to test the average difference between treatment groups. Otherwise, the Kruskal–Wallis test was performed. The LD_50_ for MCF cancer cells (MCF-7 and MCF-10A) and antioxidant activity toward ABTS and FRAP were analyzed using GraphPad non-linear regression package [log(inhibitor) vs. normalized response—variable slope]; and through a two-way ANOVA to obtain the significance values (95%, CI) of STAT3, EGFR, IGF1R kinase, CDK1, ABCC1, TGFBR1/ALK5, PARP1, ROS1 kinase, and iNOS expression. We also conducted a one-way ANOVA for miR-7-5p and continued with Tukey’s multiple comparisons test to determine further significance values in pairs of groups.

## 3. Results

### 3.1. List of Compounds from Metabolomic Profiling

The observed SBT-P metabolite profiles from the UPLC-ESI-MS/MS non-targeted metabolomic profiling analysis are presented in Table 1. The compounds are mainly divided into phytoestrogenic soy isoflavones and mycoestrogenic.

### 3.2. Pa Score, Toxicity Prediction, Druglikeness, and Network Pharmacology Analysis

SBT-P was used with target and breast cancer gene proteins to obtain a Pa score, toxicity prediction, drug similarity, and network pharmacology analysis to elucidate the targeting route at the molecular docking stage (Table 2). Compounds C1, C2, and C3 are among SBT-P isoflavones that may develop into therapeutic options targeting breast cancer and oxidants, according to the data presented in Table 2. These three compounds fulfill the Lipinski Rule with the information “Accepted” (Table 2) and have the potential (Pa > 0.7) to counter HIF1A expression-related signaling proteins linked to breast cancer. They also have a predicted LD_50_ value > 1000 and toxicity class > 4.

**Figure 2:**
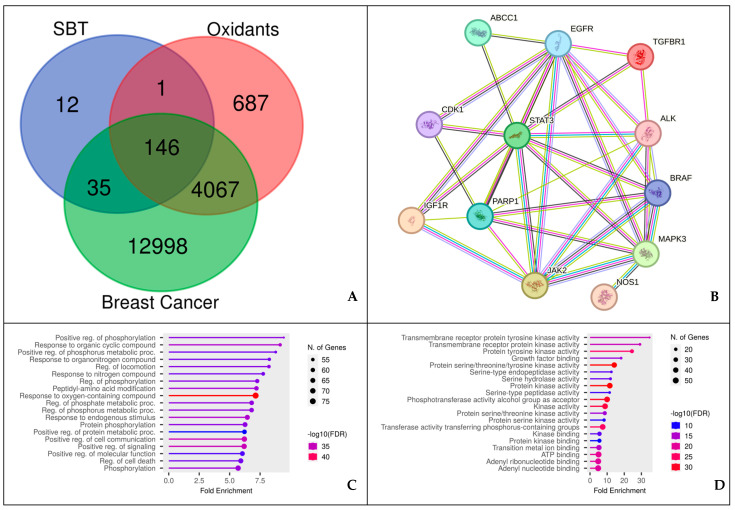

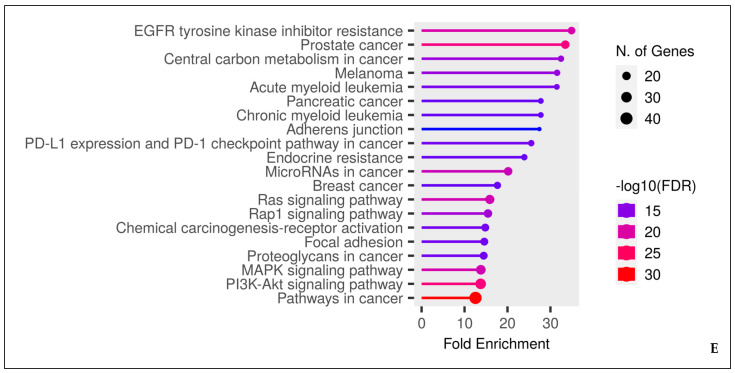
Network pharmacology of SBT-P-derived isoflavone against breast cancer. (**A**) Venn diagram displaying shared targets between SBT-P isoflavone and breast cancer-associated genes. (**B**) Protein–protein interaction (PPI) of SBT-P isoflavone targets in breast cancer. (**C**–**E**) Annotation of gene ontology (GO) biological processes and molecular function for SBT-P isoflavone targets (false discovery rate or FDR < 0.90). (**C**) GO biological processes indicating that the major SBT-P compound is a “response to oxygen-containing compounds”. (**D**) GO molecular functions indicating that the SBT-P compounds primarily target kinase activity. (**E**) KEGG pathways indicating that the SBT-P compounds primarily target cancer pathways. From figures (**C**–**E**), it appears that the network pharmacology show the benefits of the SBT-P isoflavone compound which has activity in combating oxidants or free radicals along with fighting cancer through the kinase activity pathway.

Using network pharmacology analysis, key regulators involved in cancer signaling (particularly breast cancer) were identified (Figure 2). One hundred and forty-six genes and proteins were determined as target intersections from SBT, breast cancer, and oxidants on the Venn diagram (Figure 2A). Several potential signals for cancer management were produced by an advanced analysis of the interactions between target proteins obtained from SBT and their relationship to breast cancer (Figure 2B–E). These signals include EGFR tyrosine kinase inhibitor resistance, positive regulation of phosphorylation, response to oxygen-containing compounds, regulation of cell communication, and transmembrane regulator protein tyrosine kinase activity. Table 3 displays the central regulators associated with cancer in network pharmacology (Figure 2B), which include STAT3, EGFR, IGF1R, CDK1, ABCC1, TGFBR1 (ALK5), and PARP1.

Table 3 identifies several target proteins that may be influenced by SBT. These targets encompass various processes associated with cancer development, such as oxidative phosphorylation, EGFR, HIF-1α, CDK1, ABCC1, TGFBR1, and miR-7-5p mediated through PARP1. These results indicate that SBT is engaged in multiple cancer–hypoxia and oxidant signaling pathways. Consequently, to proceed with the molecular docking simulation and include iNOS, all seven potential signaling proteins were chosen.

### 3.3. Docking Potency of SBT Compounds

Table 4 displays the results of the drug targets’ molecular docking simulation and the potency of identified SBT compounds used as molecular docking compounds with different signaling proteins as the therapeutic target. Talazoparib and S-ibuprofen were utilized as control compounds, and Table 4 displays their affinity values. All compounds exhibited better affinity values than the control for 1BG1, IM17, 3LOW, 5QIK, and 3E7G. Each compound also exhibited high affinity to specific regulators, with C3 having the highest affinity values.

### 3.4. SBT Scavenging Activity, Anticancer Capacity, and Safety

**Figure 3:**
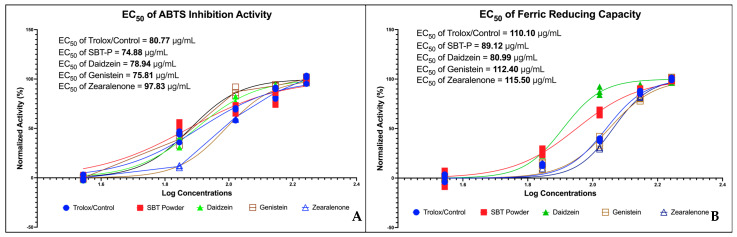
Antioxidant activity of SBT-P and SBT-P-derived isoflavone. (**A**) EC_50_ ABTS; (**B**) EC_50_ FRAP.

ABTS and FRAP EC_50_ of SBT and its compounds were compared (Figure 3). Regarding ABTS, the EC_50_ values of SBT-P, daidzein, and genistein were lower than those of trolox. However, zearalenone displayed a significantly higher value than the control. Regarding FRAP, only SBT-P and daidzein exhibited better antioxidant potential than trolox. The genistein and zearalenone EC_50_ values of ferric-reducing capacity were slightly higher than the control.

Through a two-way ANOVA, SBT-P and its isoflavone derivatives exhibited significantly lower protein expressions related to breast cancer, implying the downregulation of breast cancer signaling proteins (*p* < 0.0001). However, no significant differences were observed between SBT-P, daidzein, genistein, and zearalenone (*p* > 0.05). Therefore, SBT-P and SBT-P-derived isoflavones can inhibit breast cancer by downregulating its regulators.

**Figure 4:**
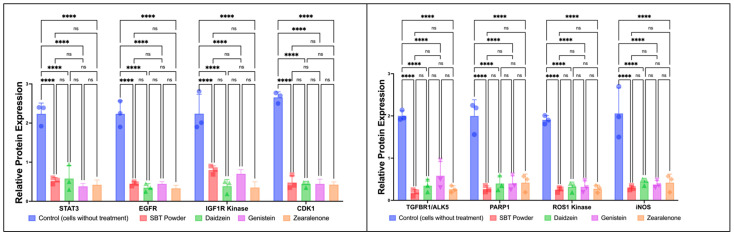
Downregulation of breast cancer signaling proteins by SBT-P and SBT-P-derived isoflavone, as evidenced on MCF-7 cells. ns, not significant, *p* > 0.05; **** *p* < 0.0001 by two-way ANOVA.

**Figure 5:**
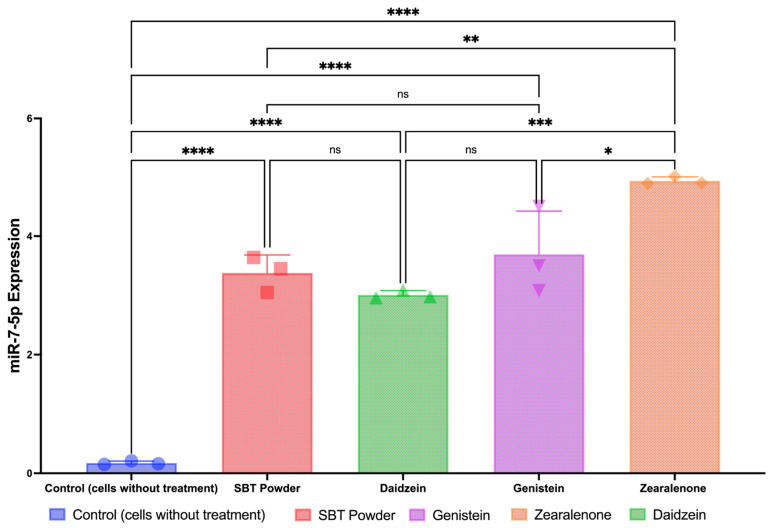
Upregulation of tumor suppressor miR-7-5p. ns, not significant, *p* > 0.05; **** *p* < 0.0001; *** *p* = 0.0004; ** *p* = 0.0023; * *p* = 0.0111 by one-way ANOVA.

In line with Figure 4, in vitro studies indicated a significant increase in the tumor suppressor miR-7-5p expression in cells treated with SBT-P and its isoflavone derivatives (*p* < 0.05; Figure 5), representing the closest possibility to causing apoptosis. However, the zearalenone-induced increase in protein expression was greater than that in other treatments, while the increased miR-7-5p expression levels after SBT-P, daidzein, and genistein treatments were similar.

The LD_50_ values of SBT-P samples on MCF-7 and MCF-10A were compared to control compounds (Table 5). The LD_50_ values of SBT-P and its isoflavone derivatives on breast cancer cells do not have a strong killing effect on MCF-7 cells. Their LD_50_ values were higher than control compounds on MCF-10A, implying the potential safety aspects of SBT-P and its isoflavone derivatives when administered as breast cancer therapy.

## 4. Discussion

A soy-rich diet has been linked to better treatment outcomes, a decreased risk of cancer recurrence after diagnosis, and a lower prevalence of different cancer forms [13,26]. Tempeh is an Indonesian food product made with soy that can cause cancer cells to undergo apoptosis and proliferate less than normal [27]. The most significant compounds in soybeans are isoflavones, phytochemicals that can function as antioxidants and shield human cells from cancer-induced oxidative stress. Other compounds associated with soybeans’ anticancer properties include phenolic compounds, saponins, phytic acid, and enzyme inhibitors like trypsin and Bowman–Birk inhibitors [10,11]. The main isoflavone in soy, genistein, inhibits cancer growth, metastasis, and development in animal models, especially by modifying genes involved in cell cycle regulation and apoptosis [13]. Furthermore, fermentation increases a food ingredient’s quantity and the activity of bioactive substances [28,29]. This phenomenon is of interest in food-based medications and technologies for developing functional foods, particularly as potential cancer-fighting prospects. Tempeh comprises bioactive peptides and isoflavones, which may help prevent cancer by acting as antioxidants or apoptotic cancer cells and inhibiting cancer cell proliferation [27,30]. An antioxidant’s function in cancer treatment is to balance ROS generation with the damage it causes to the impacted molecules (proteins, lipids, or DNA) [31]. Conversely, incorporating foods with antioxidant qualities into a dietary intervention plan improves health status [32].

Most studies have performed the metabolomic profiling of tempeh compounds [33,34]. However, many studies have not incorporated biological activity assays (in vitro and in silico antioxidant and anticancer activities). In this study, we performed a thorough multimodal in silico and in vitro analysis regarding the anticancer and antioxidant properties of SBT. Moreover, our analysis delved deeper into SBT-P and its three isoflavone derivatives. The metabolite profiling analysis revealed the presence of phytoestrogenic soy isoflavones and mycoestrogenic compounds in SBT-P. These compounds, particularly C1 and C2, demonstrated promising therapeutic potential against breast cancer, as evidenced by their favorable Pa scores, Lipinski Rule compliance, and predicted LD_50_ values. Additionally, network pharmacology analysis identified key signaling proteins associated with cancer signaling, highlighting the multifaceted engagement of SBT-P in various cancer pathways. Daidzein has been established as an anticancer and antioxidant agent while exhibiting anti-carcinogenesis, anti-fibrotic, anti-diabetic, cholesterol-lowering, and cardiovascular activities [35]. On the other hand, the prevention of cardiovascular illnesses and the reduced incidence of some cancers, particularly breast cancer, are among genistein’s principal benefits. The researchers credit this effect to the structural similarities between soy genistein and estrogen, even though the mechanism of protection against cancer involves multiple components of genistein metabolism [12].

Molecular docking simulations further supported the therapeutic efficacy of SBT-P compounds, as they exhibited higher affinity values for specific regulators than control compounds. Our in silico observations indicated an association between isoflavone and signaling pathways governing cancer cell development, suggesting distinct mechanisms. The signaling proteins targeted for binding in our investigation include STAT3, EGFR, ROS1 kinase, and iNOS, with talazoparib and S-ibuprofen selected as control substances (Table 4). Zearalenone demonstrated superior affinity to multiple regulators, indicating its potential as a lead compound for further drug development. Talazoparib has demonstrated substantial catalytic inhibition and a PARP-trapping potential roughly 100 times higher than other PARP inhibitors studied in preclinical settings [36]. The molecular docking analysis in this study revealed that nearly all SBT-P-derived metabolite constituents obtained by ultrasound and water solvents exhibited a greater affinity for the target regulators than the control, talazoparib, and S-ibuprofen. We extracted SBT-P isoflavone derivatives to assess tempeh’s antioxidant and anticancer capabilities without chemical solvents and mimic the daily consumption of tempeh by boiling or frying.

**Figure 6:**
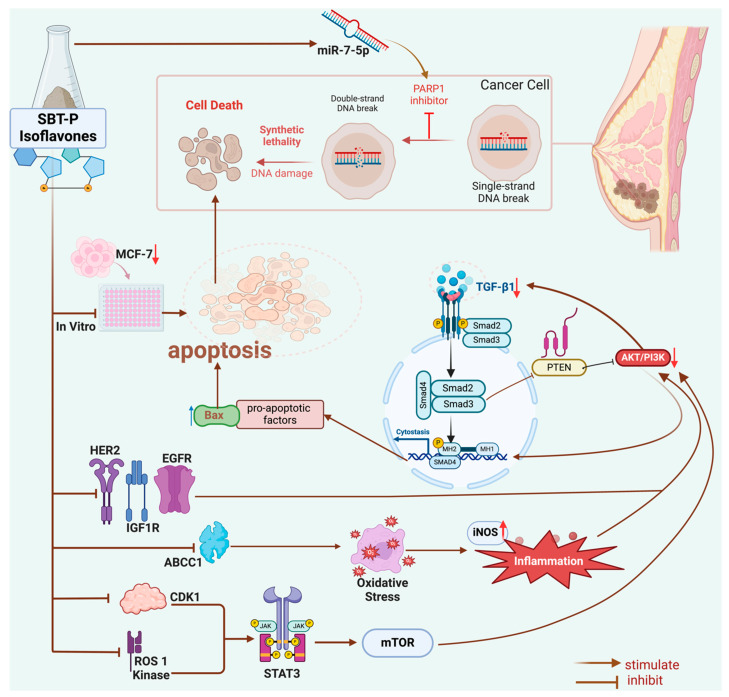
Biomechanism of SBT-P and derived isoflavones in breast cancer pathway modulation.

In vitro antioxidant assays revealed the antioxidant potential of SBT-P and its compounds, with SBT-P and daidzein exhibiting better antioxidant activity than the control. This potential may be explained by metabolites found in tempeh, such as isoflavone, daidzein, genistein, and amino acids (e.g., glutamic acid and tryptophan), which have health benefits (antioxidant, radical scavenging activity) [33]. Interestingly, a previous study highlighted that the water-soluble fraction of fermented soybean did not have significant antioxidant activities compared to tempeh [37]. Our findings imply that isoflavone derivatives may present strong antioxidant potential, as evidenced by ABTS and FRAP assays. Next, one-way ANOVA analysis indicated that SBT-P and its isoflavone derivatives significantly downregulated protein expressions related to breast cancer, supporting their role as inhibitors of breast cancer signaling proteins. Breast cancer malignancy is promoted by the signal transducer and activator of transcription 3 (STAT3), an early tumor diagnostic marker. Constitutively active and overexpressed STAT3 has a role in breast cancer metastasis, progression, and resistance to chemotherapy [38]. Isoflavones also inhibited the mechanism of cancer cell growth by inactivating the phosphoinositol 3-kinase (PI3K)/Akt/mTOR pathway [39]. The expression level of the PI3K protein, crucial for activating many proteins for cell growth and abnormal cell proliferation, decreases when cancer cells are exposed to genistein and isoflavone derivatives [8]. Furthermore, in vitro studies demonstrated the upregulation of miR-7-5p expression in cells treated with SBT-P and its isoflavone derivatives, indicating a potential mechanism underlying their anti-breast cancer effects. Although zearalenone displayed the highest increase in protein expression, SBT-P, daidzein, and genistein exhibited comparable increases in miR-7-5p expression. Previously, by altering the REGγ expression, the elevation of miR-7-5p inhibited cell proliferation and promoted cell death in breast cancer [40]. The schematic figure of the link between the anticancer mechanisms of SBT-P and its isoflavone derivatives is presented in Figure 6.

Importantly, the LD_50_ values of SBT-P and its isoflavone derivatives on breast cancer cell lines indicated their potential safety profile, as they were significantly higher than controls, implying minimal cytotoxicity toward normal epithelial cells. Natural anticancer substances can destroy malignant or altered cells without endangering healthy cells. Interestingly, because they lessen the harmful effects of the anticancer medications now on the market, natural antioxidants may help to increase patient compliance with anticancer treatment [41]. However, an equally important observation arises from comparing LD_50_ values between SBT-P isoflavone and established anticancer medications used as controls in this assay. The data reveal that SBT-P and its purified isoflavones exhibit LD_50_ values approximately 5–50 times higher than the established anticancer drugs. This discrepancy suggests a relatively weaker efficacy of SBT-P isoflavone in suppressing cancer cell survival at the studied dose levels. One plausible explanation could be differences in mechanisms of action and target specificity. While conventional anticancer drugs may directly target specific pathways or receptors implicated in cancer cell proliferation, the mechanisms underlying the anticancer effects of SBT-P isoflavone may be more nuanced and multifaceted [42]. Considering factors like bioavailability, pharmacokinetics, and the microenvironment of cancer cells, which may influence the effectiveness of SBT-P isoflavones in exerting cytotoxic effects, is also crucial.

Increased oxidative stress and unbalanced concentrations of ROS and antioxidants are characteristics of cancer; they may accelerate cancer development by triggering pro-oncogenic signaling and causing gene alterations [43,44]. Therefore, targeting oxidative stress in cancer treatment is crucial; antioxidants could substantially mitigate the incidence and progression of cancer. However, challenges persist in realizing the full potential of antioxidant therapies. Many studies employ pharmacological doses rather than dietary doses, often based solely on in vitro findings and potentially overlooking the influence of intricate in vivo conditions on antioxidant functionality. Moreover, the uneven distribution of antioxidants across different tissues and their limited bioavailability and bioaccessibility in certain organs may impede their functional efficacy. Additionally, certain antioxidants may demonstrate antioxidant or pro-oxidant properties depending on their concentration and the prevailing oxygen pressure, leading to varied outcomes upon supplementation. Importantly, most chemotherapeutic agents elevate ROS levels, thus inducing oxidative stress. Consequently, the concurrent use of antioxidants in cancer patients might counteract the intended cell death induced by chemotherapeutic drugs, resulting in an antagonistic effect [43]. In this study, it can be observed that SBT-P isoflavones, which include genistein, daidzein, and zearalenone, can molecularly act as an antioxidant through inhibiting ROS1 kinase, iNOS, ABCC1, and CDK1 so that oxidative stress does not occur in target cells (thereby reducing inflammation as in the mechanism that is depicted in Figure 6); in vitro, they also significantly inhibit iNOS and ROS1 kinase, as shown in Figure 4. Interestingly, the role of daidzein is more clearly confirmed in vitro as it can scavenge ABTS and FRAP free radicals better than control/trolox, and this is seen in Figure 3. The findings presented in this study support the potential of SBT-P and its isoflavone derivatives as promising therapeutic agents for treating breast cancer. Further investigation into their mechanisms of action and clinical efficacy is warranted to validate their therapeutic utility in clinical settings.

As a strength of this study, the results of the research highlight the complex nature of the interactions between the SBT-P isoflavones and the protein receptor targets, which have never been previously reported. The SBT-P isoflavones have significant potential in anticancer therapeutic and antioxidative applications due to their capacity to bind to various targets with different molecular affinities. This is particularly advantageous in the treatment of complicated cancer-related–oxidative disorders that require a multifaceted approach. Additionally, this study examines the possible synergistic effects of SBT-P isoflavones that may be used to enhance the efficacy of therapeutic treatments in human breast cancer. In addition, combining in silico studies with in vitro studies can provide new evidence-based insights into anticancer benefits. As an additional note for the discussion of Figure 4 and Figure 5, and as a limitation of the study, each of the protein and gene expression values were not normalized by an internal control; this is due to our small sample size. With a small sample size, including internal controls for normalization may introduce noise or reduce statistical power. In such instances, researchers may choose to omit normalization to avoid potential biases. In certain experimental designs, researchers may be more interested in comparing relative changes in gene/protein expression between different conditions rather than absolute expression values. In such cases, normalization may not be considered essential.

## 5. Conclusions

This study provides a comprehensive analysis of the therapeutic potential and safety profile of SBT-P and its derived isoflavones in managing breast cancer. Metabolomic profiling revealed a rich composition of phytoestrogenic soy isoflavones and mycoestrogenic compounds in SBT-P, indicating its potential as a source of bioactive agents. Through combining in silico analysis, network pharmacology, and molecular docking, key regulators involved in breast cancer-related oxidant signaling were identified, highlighting the potential of SBT-P compounds—particularly daidzein and genistein—as both therapeutic options targeting breast cancer and antioxidant agents. In vitro studies further support the anti-breast cancer and radical scavenger effects of SBT-P and its isoflavone derivatives, revealing significant downregulation of breast cancer regulators and an increased expression of miR-7-5p, a tumor-suppressive microRNA. Importantly, the LD_50_ values of SBT-P and its derivatives on breast cancer cell lines indicated their potential safety, suggesting minimal cytotoxic effects on MCF-10A cells compared to controls, thus underscoring their promising safety profile for breast cancer therapy. Overall, these findings provide valuable insights into the therapeutic potential of SBT-P and its derived compounds in managing breast cancer, warranting further investigation and clinical exploration for their translation into effective therapeutic interventions.

## Figures and Tables

**Table 1:** SBT-P compound-derived isoflavone.

No	Type	Name	Molecular Formula	PubChem/CAS ID	*m*/*z*	^t^R
C1	Phytoestrogenic	Daidzein	C_15_H_10_O_4_	5281708/486-66-8	254.2000	2.69
C2	Soy isoflavones	Genistein	C_15_H_10_O_5_	5280961/446-72-0	270.2395	2.81
C3	Mycoestrogenic	Zearalenone	C_18_H_22_O_5_	5281576/17924-92-4	318.3900	1.98

^t^R, retention time.

**Table 2:** Anticancer potential evaluation of SBT-P-derived isoflavones based on SAR predictions, Pa score, toxicity prediction, druglikeness, and network pharmacology analysis.

Code	Pa Score *	Toxicity Model Computation Analysis **		Druglikeness ***		
	HIF1A Expression Inhibitor	Predicted LD_50_ (mg/kg BW)	Toxicity Class	Lipinski Rule	Pfizer Rule	GSK
C1	0.915	2,430	5	Accepted	Accepted	Accepted
C2	0.939	2,500	5	Accepted	Accepted	Accepted
C3	0.871	500	4	Accepted	Accepted	Accepted

* Way2Drug; ** Protox; *** ADMET.

**Table 3:** Results of the top one PPI network analyses.

Name	Degree	Betweenness Centrality	Closeness Centrality	Overall Score	Pathway
STAT3	19	0.50293053	0.52054795	20.0234785	Immune cells, and adipocytes in the tumor microenvironment, Warburg effect, oxidative phosphorylation, STAT3 signaling regulates glycolysis, ROS production or ROS1 kinase, lipid, and glutamine metabolism in cancer cells
EGFR	14	0.12136164	0.43428571	14.5556474	EGFR promotes NF-kappa-B activation and cell proliferation, ALK, and ROS1 mutations
IGF1R	6	0.0216914	0.38383838	6.40552979	PI3K/AKT and MAPK signaling pathways and HIF-1α (oxygen-sensitive subunit of HIF1) signaling
CDK1	4	0.0174118	0.32900433	4.34641613	CDK1 mediates the activation of the FBXO28 ubiquitin ligase and promotes MYC-driven transcription and tumorigenesis
ABCC1	1	1	1	3	ABCC1, ABCC4 play an essential physiological role in its development, independent of their role in multidrug resistance, affecting, migration cellular proliferation, and differentiation
TGFBR1 (ALK5)	1	1	0.3220339	2.3220339	Poly(ADP-ribose) polymerase-1 (PARP-1) regulates TGF-β receptor I (TβRI) expression and ALK5/SMAD2/3 signaling in regulating NOX expression
PARP1	2	0.02631579	0.28358209	2.30989788	MiR-7-5p-mediated downregulation of PARP1 impacts the repair of DNA homologous recombination, NF-κB signaling pathway, and the inhibition of poly(ADP-ribose) polymerase (PARP) activity induces synthetic death in mutated BRCA1/2 cancer

**Table 4:** ΔG of molecular docking parameter of SBT-P-derived isoflavone.

Receptors/Proteins (PDB ID)	Gibbs Free Energy (ΔG; kcal/mol)				
	C1	C2	C3	Control/Talazoparib	Control/S-ibuprofen
STAT3 (1BG1)	−8.1	−8	−7.1	−7	
EGFR (1M17)	−8.2	−7.7	−7.5	−7.4	
IGF1R kinase (3LWO)	−8.8	−8.6	−9.4	−8.5	
CDK1 (6GU7)	−7.6	−7.6	−8.4	−8.2	
ABCC1 (2CBZ)	−6.7	−6.9	−7.1	−6.7	
TGFBR1/ALK5 (5QIK)	−9.3	−9.7	−7.5	−7.2	
PARP1 (4R6E)	−8	−8.1	−9.6	−8.4	
ROS1 kinase (3ZBF)	−7.1	−7.1	−8.1	−8	
iNOS (3E7G)	−9.8	−9.8	−8.8		−8.6

**Table 5:** LD_50_ values (μg/mL) on MCF-7 and MCF-10A lines.

No	Samples	MCF-7 Cancer Line	MCF-10A Normal Line
1	SBT-P	419.0045	1995.5099
2	Genistein	50.7880	1065.0034
3	Daidzein	45.8998	998.6090
4	Zearalenone	39.0887	1010.4661
5	Control mitoxantrone	21.0555	18.0950
6	Control talazoparib	8.0905	20.1908

## Data Availability

The datasets presented in this study can be requested from the corresponding author or F.N.

## Supplementary Materials

The following supporting information can be downloaded at: https://www.mdpi.com/article/10.3390/antiox13060632/s1, Table S1. SMILES Canonical for SBT-P Metabolites.
