# Supplementary material for: Mechanism of Action of Isoflavone Derived from Soy-Based Tempeh as an Antioxidant and Breast Cancer Inhibitor via Potential Upregulation of miR-7-5p: A Multimodal Analysis Integrating Pharmacoinformatics and Cellular Studies
Source: Antioxidants (Basel). 2024 May 22;13(6):632. doi: 10.3390/antiox13060632 (PMC11200984; doi:10.3390/antiox13060632)
Supplement: Supplementary file 1 [file antioxidants-13-00632-s001.zip › antioxidants-2967697-supplementary.pdf]

Smiles Canonical SBT

Compounds	SMILES
C1	C1=CC(=CC=C1C2=COC3=C(C2=O)C=CC(=C3)O)O
C2	C1=CC(=CC=C1C2=COC3=CC(=CC(=C3C2=O)O)O)O
C3	CC1CCCC(=O)CCCC=CC2=C(C(=CC(=C2)O)O)C(=O)O1
